# New products

**DOI:** 10.1155/S1463924697000047

**Published:** 1997


Journal of Automatic Chemistry, Vol. 19, No. 1 (January–February 1997) pp. 27–29

### New products

### Titroprocessor

The 726 Titroprocessor is the latest member of the Metrohm titrator family. It performs kinetically slow reactions, such as diazotizations and nonaqueous titrations, as well as fast reactions, such as acid-base titrations. In addition to this it also performs end-point and Karl Fischer titrations (moisture content), plus measures pH, U/mV and temperature and it can be used as an independent dosing unit.

Results and methods for back titrations, determinations of pK or HNP (half neutralization potential), amperometric titrations, and complex dosing regimes can be stored within the instrument or on a memory card (SRAM card). To facilitate rapid use of the instrument after installation, each instrument comes with a read-only memory card containing a large number of tried and tested methods.

The 726 Titroprocessor is available with one or two sets of measuring inputs, each set comprising two inputs for high impedance electrodes, one for the temperature sensor and one for the polarized electrode. There are also four connections for dosing instruments which can be used as titrating or auxiliary burettes. Two RS 232 interfaces ensure quick communication with the outside world.

On the LCD screen, titration curves in real time, measuring point lists, volume/time curves as well as superimposed curves can be displayed. The print-out according to GLP occurs either on the integrated printer or if preferred an external device.

*For further information telephone Metrohm UK Ltd on* 01280 824 824.

### Combinatorial chemistry

An application note (No. 222/ICA Version 1) is now available from Micromass Ltd on *High Throughput LC-MS Verification of Drug Candidates from 96-Well Plates.* With the advent of combinatorial chemistry, the pharmaceutical industry has radically altered its approach to drug discovery. The aim is to vastly increase the number of potentially useful organic compounds synthesized, with many more compounds being screened for biological activity, resulting in more drugs being found more quickly. This stands in contrast to the traditional situation of labour-intensive, step-wise synthesis of individual compounds and their analogues, which would be followed by meticulous isolation, purification and characterization.

Covering several quite different concepts, combinatorial chemistry originally evolved out of the necessity to generate a diverse range of large numbers of organic compounds, the structures of which can be guided by computer-aided design. The technique has three basic methods, for which libraries have been created, consisting of either individual compounds in separate vessels or an entire mixture of reaction products. These libraries are then screened for biological activity, and any indication of a positive response results in the isolation, identification and characterization of a compound. Its efficacy is then studied by drug metabolism and pharmacokinetic studies.

As the approach to the synthesis of drug candidates has had to change to meet the high numbers of compounds required, so has the approach to compound characterization. Previously, when limited numbers of organic compounds were individually synthesized, each was

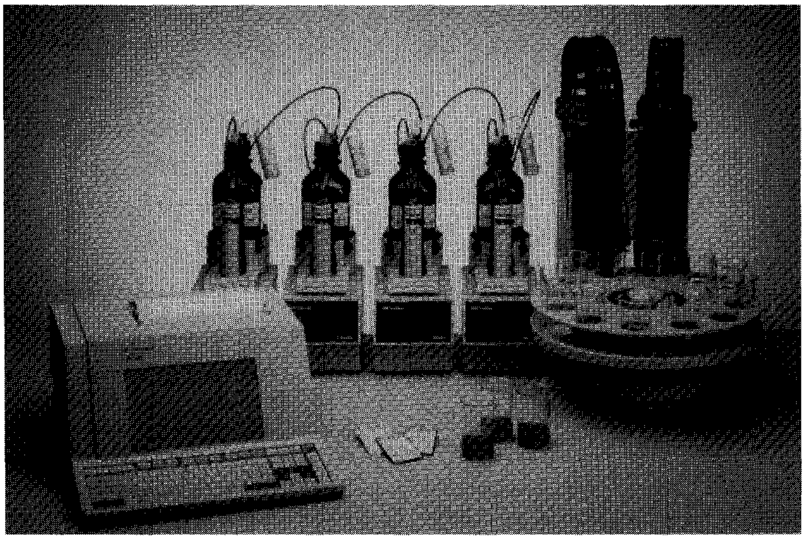

*The 726 Titroprocessor from Metrohm connected to the company's 717 Sample changer. See text for details.*

0142-0453/97 $12·00 © 1997 Taylor & Francis Ltd completely characterized using multiple analytical methods. These included melting point determination, combustion analyses, infra-red spectrophotometry, ultraviolet spectrophotometry, nuclear magnetic resonance spectroscopy, and mass spectrometry.

The increase in the number of drug candidates has made the complete analysis process far too time-consuming and perhaps even unnecessary at the time of initial synthesis, as the majority of compounds synthesized do not exhibit suitable biological activity. A new analytical technique is needed, to state categorically—'yes' or 'no'—and confidently whether any of the expected synthetic products are present at the end of the syntheses.

Atmospheric Pressure Ionization (API) mass spectrometry is the most suitable analytical technique capable of meeting these criteria. It can be applied with equal ease to both single and multi-component samples, the latter of which may require on-line liquid chromatography (LC), and it also generates unambiguous molecular weight information for the majority of organic compounds. The application note describes OpenLynx-Diversity, which integrates a sample handling system for 96-well plates, an LC procedure, and a bench-top API mass detector within a Microsoft Windows-NT environment.

*For further information contact Dr Andrew Eaton, Micromass Ltd, Floats Road, Wythenshawe, Manchester, M23 9LZ, UK. Tel.: 0161 945 4170; fax: +44 0161 998 8915; E-mail: andrew.eaton@micromass.co.uk.*

### Titrator

Until now the Titrino titrators were stand-alone instruments; the 721 NET Titrino was designed for PC linkage, and does not have separate keypads. Methods, parameters, as well as evaluations and calculations are entered via the user's PC. The system fulfills the requirements of ISO and GLP, which demands that all settings and results are stored for several years. The 721 NET Titrino has five operating modes: dynamic titration; monotonic Titration; titration to a fixed endpoint; measurement of pH, voltage or temperature; and pH calibration with up to nine buffers.

*For further information telephone Metrohm UK Ltd on 01280 824824.*

### Materials checking

Perkin-Elmer has introduced a system for materials checking and assay determination: the IdentiCheck FT-NIR systems. The combination of an FT-NIR spectrometer, unique sampling options and validated software provides a system which can be optimized to meet specific operating requirements.

The systems are an alternative to traditional dispersive NIR instruments, covering the first and second overtone regions. Utilizing the full first overtone region, which extends beyond 2,500 nm, improves results and allows new applications and sampling opportunities. Two systems are available. The Spectrum Identi-Check FT-NIR System comprises the IdentiCheck FT-NIIR Spectrometer and Spectrum IdentiCheck Software for Windows-based, PC-controlled operation. The Paragon IdentiCheck FT-NIR is a fully-integrated system comprising the IdentiCheck FT-NIR Spectrometer and paragon IdentiCheck Software. Both systems include flexible and versatile sampling accessories. A reflectance accessory allows analysis of powders and tablets, for example, in their containers, eliminating sample preparation. A remote sampling interface offers remote fibre-optic sampling of liquids and solids and can be installed simultaneously with the standard transmission and reflectance systems.

*For further information, contact Perkin-Elmer Ltd, Post Office Lane, Beaconsfield, Buckinghamshire HP9 1QA, UK. Tel.: 01494 676161; fax: 01494 679331/3. E-mail http://www.perkin-elmer.com.*

### FT-IR microscopy

Perkin-Elmer's AutoIMAGE System integrates a new microscope design with the company's IMAGE software. All microscope operations, including adjustments to aperture, focus and illumination, are automated and controlled from a panel on the PC screen. The user also has access to new modes of operation, such as automatic ATR mapping for surface studies.

Features such as Auto View, Auto Status, Auto Marker, Auto Correlation, Auto Illumination and Auto Mode save analysis time; other key features of the AutoIMAGE System include Auto Focus, which allows refocusing during unattended data collection operations such as line scanning or mapping, and Auto Aperture, which enables users to interactively select the sampling area on the visible image. Simultaneous Variable Magnification (SVM) provides users with access to any magnification view of the sample. The infra-red mapping area is defined simply by drawing a moveable growbox on the video image using the mouse. The AutoIMAGE Microscope provides superior IR performance, spatial resolution and visible image quality. With all functions controlled via an interactive panel on the PC screen, conventional controls are eliminated for simplicity and speed of operation.

*For further information contact Perkin-Elmer Ltd (as before).*

### 'FAST' interactive training

Thermo Jarrell Ash Corporation have launched their FAST (Fully Interactive Atomic Spectroscopy Training) software on CD ROM for all their spectroscopy products. FAST software gives instant access to text and video clips (with sound) explaining the routine operation and maintenance of the instrument and accessories, as well as instrument theory.

FAST is a new concept in training designed to provide the operator with virtually all of the information normally given during an intensive training course. However, FAST allows training at the operator's convenience, and can instantly access specific bits of information pertaining to immediate topics of interest. FAST is normally resident on the instrument's computer and operates in the Windows environment. Therefore, immediate visual and spoken help is available for adjustments or optimization parameters that would normally require an operator's manual and/or a phone call.

*For more information contact Thermo Jarrell Ash, 27 Forge Parkway, Franklin, MA 02038, USA. Tel.: 508 520-1880; fax: 508 520 1732.*

### Benchtop fluorine analysis

Oxford Instruments' Industrial Analysis Group's QP20+ is the latest generation of the QP20. These use pulsed Nuclear Magnetic Resonance (NMR) to measure a wide range of components, including water, oil, fats, and chemical products, usually in less than 30 seconds. A new application package determines fluoride in toothpaste, fluorine in waste solvents and in alumina smelting scrubbers.

The analyser now features an optional PC operating system, running under MS Windows. The user is offered routine operation by simple keyboard or mouse functions, a large clear display of individual results or tabular displays of a series of results, fully integrated with MS Excel™ for customized data presentation, and a flexible Pulse Sequence Editor. The analyser is also available without a PC for simple, routine checks of production samples as a console operation system.

*Further information from Oxford Instruments' Industrial Analysis Group, 130A Baker Avenue Ext., Concord, MA 01742, USA; tel.: 800 447 4717 or 508 371 9009; or 19/20 Nuffield Way, Abingdon, Oxon OX14 1TX, UK; tel.: 01235 532, 123, fax (01235) 535 0416.*

### Alternative solvents

Following similar developments in the USA and Japan, approvals have now been received for pre-marketing a new range of DuPont cleaning and drying agents in the European Union. The products, to be marketed under the Vertrel brand, are near 'drop-in' replacements for ozone depleting CFC-based solvents formerly widely used in the electronics and metal cleaning industries and being phased out under the Montreal Protocol.

The Vertrel® product line is designed to offer an alternative to users currently seeking long-term replacements for halogenated solvents such as CFC-113, 1.1.1 trichloroethane; and for HCFCs such as HCFC-225 ca/cb and HCFC-141b, regulated under the Montreal Protocol. They can also be considered as alternatives for PFCs (perfluorocarbons) in some applications where these have been adopted because of the lack of timely alternatives to CFC-113.

The new products are designed to be used in existing equipment, subject to certain DuPont recommended modifications to minimize emissions. They are suitable for removal of a large spectrum of contaminants in the field of printed circuit board assembly, metal cleaning, decontamination, displacement drying prior to metalization, high precision cleaning, and also as carrier fluids for lubricant deposition or as dielectric fluids.

Currently manufactured in the company's Ponca City plant in Oklahoma, US, the products will be shipped to Europe from a new plant recently started up at the DuPont Mitsui Shimizu fluorochemicals complex in Japan.

*Further information from Tim Horsten, Fluorochemicals, DuPont International, Geneva, Switzerland. Tel.: +41-22-717 50 81; fax: +41-22-717 61 69.*

